# Is Electroacupuncture an Effective and Safe Treatment for Poststroke Depression? An Updated Systematic Review and Meta-Analysis

**DOI:** 10.1155/2021/8661162

**Published:** 2021-08-24

**Authors:** Xiafei Wang, Wa Cai, Yongpeng Wang, Song Huang, Quanbin Zhang, Feng Wang

**Affiliations:** ^1^Departments of Neurology, Seventh People's Hospital of Shanghai University of Traditional Chinese Medicine, Shanghai, China; ^2^Department of Acupuncture, Shanghai Shuguang Hospital Affiliated to Shanghai University of Traditional Chinese Medicine, Shanghai, China; ^3^Department of Radiology, Seventh People's Hospital of Shanghai University of Traditional Chinese Medicine, Shanghai, China; ^4^Department of Neurosurgery, Shanghai Tenth People's Hospital, Tongji University School of Medicine, Shanghai, China

## Abstract

**Objective:**

To observe and compare the efficacy and safety of electroacupuncture and antidepressants in the treatment of poststroke depression (PSD) using a meta-analysis method.

**Methods:**

The VIP, CNKI, Wanfang, CMB, Embase, PubMed, and Cochrane databases were searched. All randomized controlled trials (RCT) on electroacupuncture treatment of PSD were searched and further screened. Meta-analysis was performed on electroacupuncture and western medicine for PSD to explore the difference in efficacy between electroacupuncture and western medicine for PSD.

**Results:**

Nineteen RCTs were included in the meta-analysis. Compared with the Western medicine group, the meta-analysis showed no significant changes in Hamilton Depression Scale (HAMD) scores between the electroacupuncture group and the antidepressant group (*P* > 0.05). The number of adverse events in the electroacupuncture group was less than that in the antidepressant group.

**Conclusion:**

Compared with antidepressants, electroacupuncture is not less effective in improving depression symptoms in PSD patients with greater safety.

## 1. Introduction

Poststroke depression (PSD) is the most common complication of poststroke affective disorder. It has been a major health issue due to its detrimental effects on cognitive function, social activity, and stroke rehabilitation [1]. PSD is the focus of stroke treatment and prevention in China. As one of the traditional therapies in China, electroacupuncture (EA) has been demonstrated to be effective in the treatment of PSD in a couple of clinical studies [2–4]. The early intervention with EA was shown to be beneficial for subsyndromal depression, with significantly improved symptoms and quality of life after 6 weeks of treatment [5]. With the development of evidence-based medicine, more and more randomized controlled trials (RCTs) of electroacupuncture therapy come out gradually. However, its imperfect methodology results in low study quality. Currently, there have been many RCTs on electroacupuncture treatment of PSD that demonstrated that acupuncture treatment of PSD has definite efficacy and fewer side effects. Therefore, this study will figure out the differences in efficacy and safety between electroacupuncture and antidepressant treatment for PSD based on meta-analysis to determine the advantages of electroacupuncture compared with antidepressant treatment.

## 2. Methods

### 2.1. Literature Search

#### 2.1.1. Search Scope

The VIP, CNKI, Wanfang, CMB, Embase, PubMed, and Cochrane databases were searched. The deadline for literature search was on September 30, 2020.

#### 2.1.2. Search Terms

The following are the search terms:

1. Define disease: stroke, stroke, depression (post stroke, post cerebral hemorrhage, post cerebral ischemia, depressive disorder, depressive)
2. Definition of intervention measures: Electroacupuncture, Electroacupuncture, Electrical acupuncture
3. Definition of study type: controlled clinical trial, randomized controlled trial, randomized trial
4. “OR” is used between two search terms with the same or similar definitions. “AND” is used between multiple search terms with different definitions

### 2.2. Inclusion Criteria

The following are the inclusion criteria:

1. RCT literatures are in English and Chinese on electroacupuncture treatment of PSD
2. The subjects were patients with PSD who met the diagnostic criteria of stroke and depression
3. The experimental group was treated with electroacupuncture as the only treatment, whereas the control group was treated with antidepressants as the only treatment
4. The main outcome measure is Hamilton Depression Scale (HAMD)

### 2.3. Exclusion Criteria

The following are the exclusion criteria:

1. Animal researches, case reports, reviews, and commentary or evaluative researches
2. Studies about nonstroke depression, such as primary depression
3. Repeatedly published studies
4. Studies where full literature is not available

### 2.4. Literature Screening

Two researchers independently completed the literature screening work. First, titles and abstracts of all studies were collected. Unrelated studies were excluded by preliminary screening. Then, full texts of remaining literatures were reviewed. Those that met the criteria were selected for inclusion in the final study. The decision was reached through discussion if there were different opinions.

### 2.5. Data Extraction

A predetermined data collection form was designed including the following information:

1. General information: name of author, publication year, sample size, sex, age, intervention methods, course of treatment, frequency, outcomes, adverse events, and follow-up
2. Methodological characteristics: method of randomization, blindness and allocation, data integrity, and selective reporting

### 2.6. Quality Assessment

The methodological quality of the included literatures was assessed using the modified Jadad scale, which included four aspects:

1. Randomization

- Appropriate: computer-generated randomization or similar randomization method (2 points)
- Unclear: lack of detailed descriptions of randomization method (1 point)
- Inappropriate: unscientific method of randomization (0 point)

(2) Hiding of randomization

- Appropriate: methods such as use of sealed opaque envelopes with serial numbers (2 points)
- Unclear: the use of random number table or other methods (1 point)
- Inappropriate: alternative assignment, case number, or special dates that increase the possibility of unhiding (0 point)
- Not used (0 points)

(3) Blindness

- Appropriate: use of placebo or similar methods (2 points)
- Unclear: lack of detailed descriptions of blindness (1 point)
- Inappropriate: inappropriate method of blindness (0 point)

(4) Withdrawal

- The reasons for withdrawal which were described (2 points)
- Lack of description of the reasons for withdrawals (1 point)

Two researchers independently evaluated and rated the quality of the include literatures. A score of 1 to 3 indicated low quality, while a score of 4 to 7 indicated high quality.

#### 2.6.1. Heterogeneity Assessment

Heterogeneity in the included studies was assessed by *I*^2^ values. If *I*^2^ < 50%, there was no obvious heterogeneity. If *I*^2^ ≥ 50%, the heterogeneity was higher.

### 2.7. Subgroup and Sensitivity Analysis

If the heterogeneity was high (*I*^2^ ≥ 50%), studies that might lead to heterogeneity were excluded by sensitivity analysis. If the heterogeneity remained high, factors that may lead to heterogeneity were determined; then subgroup analysis was conducted according to these factors.

### 2.8. Assessment of Publication Bias

If more than 10 studies were included, funnel plots were used to assess publication bias.

### 2.9. Statistical Analysis

Meta-analysis was performed using Review Manager V.5.3 software. Continuous variables were presented as the mean difference (MD), whereas dichotomous variables were presented as relative risk ratios (RRs) with 95% confidence intervals (CIs). If there was no significant heterogeneity (*I*^2^ < 50%), a fixed effects model was used. The random effects model was used in case of significant heterogeneity (*I*^2^ ≥ 50%). *P* < 0.05 indicates that the difference is statistically significant.

## 3. Results

### 3.1. Literature Search

**Figure 1:**
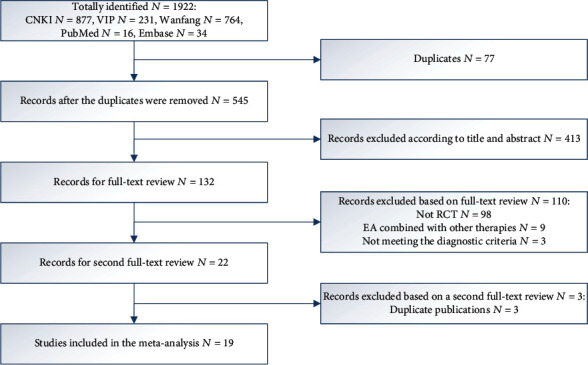
Flow chart of literature selection.

A total of 1922 records were obtained through electronic databases. 19 RCTs were included in the meta-analysis. The process of literature retrieval and screening is presented in Figure 1.

### 3.2. Characteristics of Included Trials

All the studies included in the meta-analysis were randomized, parallel, controlled trials. Of the 19 studies included, a total of 848 patients were in the electroacupuncture group with 758 patients in the control group. Patients in 18 studies were diagnosed with stroke according to Chinese Classification of Cerebrovascular Diseases (CCCD of 2015 edition), while patients in only one study were diagnosed by CT or MRI. The diagnosis of depression was based on Diagnostic Criterion for Mental Disorders (3rd Edition) (CCMD-3) in 15 studies, and the severity of depression was assessed by Hamilton Depression Scale (HAMD) in 19 studies. The course of electroacupuncture treatment was 4-12 weeks, and the treatment frequency ranged from 20 to 45 times. The average number of selected acupoints was 4.1. The two most frequently used acupoints were GV20 and EX-HN3. Among them, GV20 was selected in 13 studies [6–18], and EX-HN3 was selected in 8 studies [7]. Use of antidepressants included fluoxetine (10-40 mg/day), paroxetine hydrochloride (20 mg/day), citalopram (20 mg/day), and sertraline hydrochloride (50 mg/day). Patients in the control group in 16 studies [2, 4, 6–19] were administered with fluoxetine. HAMD was used in all the included studies to assess the change of depression severity. Of them, one study [12] used the TESS score to evaluate adverse events. The general characteristics of all the included studies are shown in Table 1.

C: control group; CCCD: Chinese Classification of Cerebrovascular Disease; CCMD: Chinese Classification and Diagnostic Criteria of Mental Disorders; HAMD: Hamilton Depression Scale.

### 3.3. Quality Assessment

**Figure 2:**
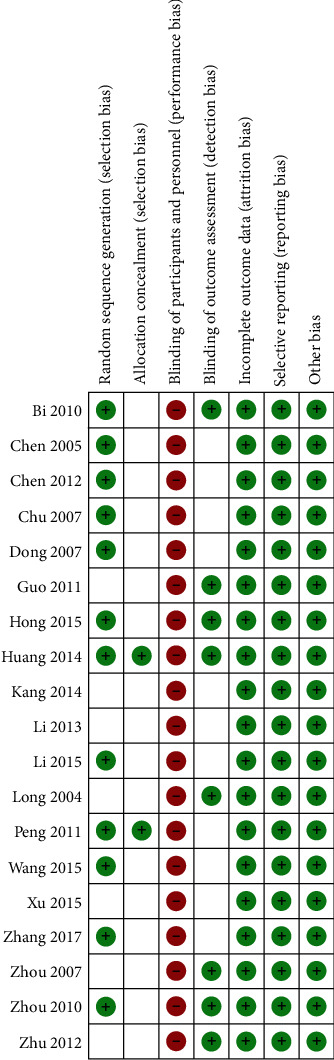
Risk of bias summary.

All 19 studies were claimed to be randomized, but 5 studies [2, 9, 12, 15, 19] did not state the method of random sequence generation. Two studies [7, 11] reported the procedure for allocation concealment. Nine studies [1, 3, 5, 7, 10, 11, 13, 16, 17] used the random number table method. Three studies [6, 8, 14] were randomly grouped according to the order of admission or treatment. Two studies [4, 18] used computer software to randomly generate random numbers and groups for grouping. None of the studies blinded the control group patients, and only 8 studies [5–7, 9, 13–15, 19] mentioned blinding of outcome assessment. In two studies [7, 11], there are three dropouts. In one study [13], seven patients dropped out. The loss rate of all the studies was less than 5%, which did not affect the statistics of the results, so the above thirteen patients were excluded. The Jadad scores of each study are shown in Table 1, and the assessment of bias risk of each study is shown in Figure 2.

### 3.4. Results of Meta-Analysis

#### 3.4.1. HAMD Score

**Figure 3:**
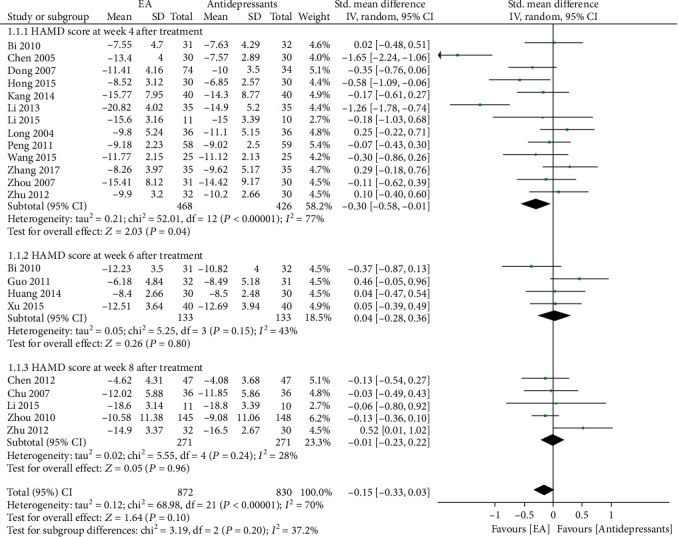
Forest plot of electroacupuncture versus antidepressants: Hamilton Depression Rating Scale (HAMD) score at different endpoints.

*(1) Meta-Analysis*. HAMD was used by all studies to assess the severity of depression. The endpoints of outcome assessment were weeks 4, 6, and 8 after treatment (Figure 3). Thirteen studies [1, 3–6, 8, 9, 11, 14–16, 18, 19] recorded HAMD scores before and after week 4. Meta-analysis showed that HAMD scores were significantly reduced in the electroacupuncture group compared to the antidepressant group (P&L; 0.01; SMD -0.30, 95% CI: -0.58, -0.01) but showed high heterogeneity (*X*^2^ = 52.01, *I*^2^ = 77%). Four studies [2, 7, 12, 14] recorded HAMD scores at week 6 before and after treatment. Meta-analysis showed no significant difference in HAMD scores in the electroacupuncture group compared to the antidepressant group (*P* = 0.15; SMD 0.04, 95% CI: -0.28, 0.36). Five studies [4, 9, 10, 13, 17] recorded HAMD scores at week 8 before and after treatment. Meta-analysis showed that HAMD scores did not change significantly in the electroacupuncture group compared to the antidepressant group (*P* = 0.24; SMD -0.01, 95% CI: -0.23, 0.22).

**Figure 4:**
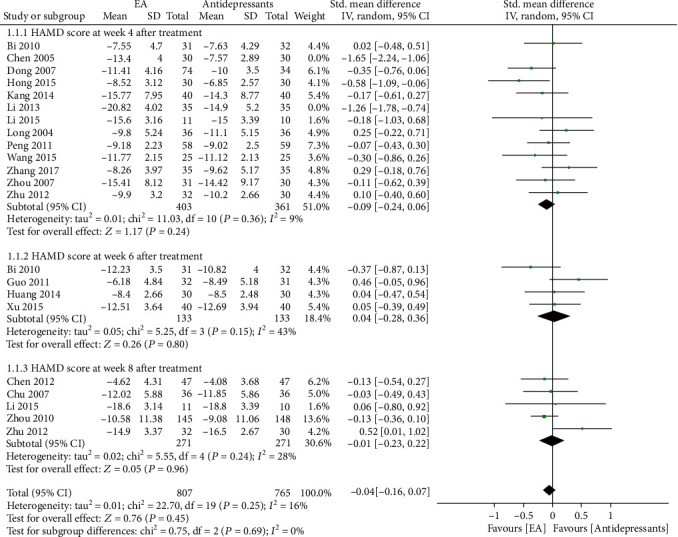
Sensitivity analysis: Hamilton Depression Rating Scale (HAMD) score at different endpoints.

*(2) Sensitivity Analysis*. HAMD scores at week 4 before and after treatment were recorded in thirteen studies, and the meta-analysis was highly heterogeneous (*I*^2^ = 77%), so sensitivity analysis was conducted. According to the principle proposed by Patsopoulos et al. [20], it was found that two of the studies [8, 18] had the greatest influence on heterogeneity which was significantly reduced after the deletion of these two studies. However, compared with the antidepressant group, the HAMD score in the electroacupuncture group did not change significantly (*I*^2^ = 9%, *P* = 0.36; SMD -0.09, 95% CI: -0.24, 0.06, Figure 4).

#### 3.4.2. Adverse Events

**Figure 5:**
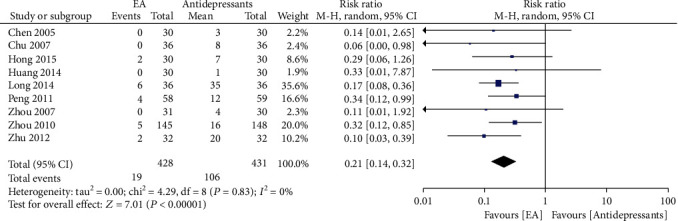
Forest plot of electroacupuncture versus antidepressants: incidence of adverse events.

Nine studies [5, 7, 9, 11, 13, 15, 17–19] reported adverse events (AEs) during treatment (Figure 5). In the EA groups, the most commonly recorded AEs were fainting during acupuncture treatment, subcutaneous haemorrhage, pain and nausea. AEs occurring in the antidepressant groups included dry mouth, dizziness, somnolence, constipation, nausea, anorexia, diarrhoea, and headache. Meta-analysis showed that EA treatment was associated with significantly fewer AEs when compared with antidepressants (RR 0.21, 95% CI: 0.14, 0.32) and no heterogeneity (*Χ*^2^ = 4.29, *I*^2^ = 0%).

#### 3.4.3. Publication Bias Analysis

**Figure 6:**
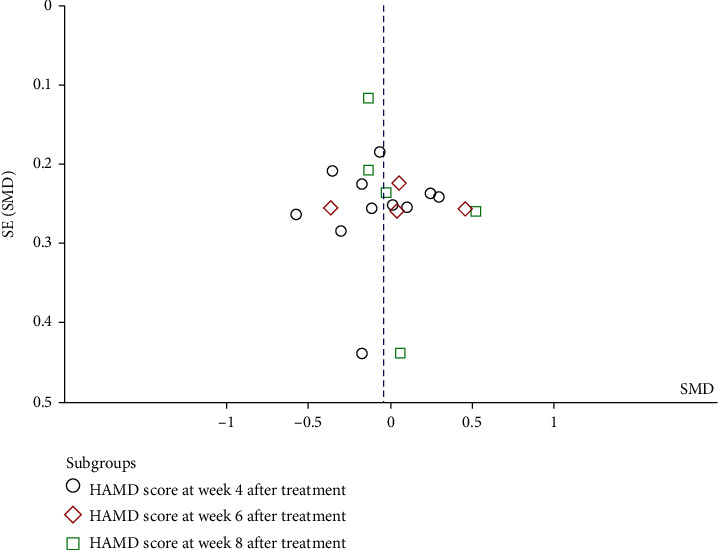
Funnel plot of electroacupuncture versus antidepressants: Hamilton Depression Rating Scale (HAMD) scores at different endpoints.

The funnel plot of the HAMD score at different endpoints (Figure 6) showed no asymmetry, indicating that the 19 included studies had no evidence of significant publication bias. A funnel plot of the incidence of AEs was not generated due to the fact that the number of included studies is less than 10.

## 4. Discussion

As an important indicator to evaluate the status of depression, accurate measurement of HAMD score is of great significance to the diagnosis and treatment of depression. Currently, the effectiveness of HAMD score has been taken as an outcome indicator by a large number of studies. All studies included in this meta-analysis used changes in HAMD score as the primary outcome indicator of results. All included studies used the HAMD scale to assess depressive symptoms. We evaluated the improvement of depressive symptoms after 4, 6, and 8 weeks of treatment. Among them, HAMD scores were evaluated at week 6 and week 8 after treatment. Meta-analysis showed that there were no significant changes of the HAMD score in the EA group compared to the antidepressant group. Thirteen studies recorded HAMD scores at week 4 before and after treatment. Meta-analysis showed a significant reduction in HAMD scores in the EA group compared to the antidepressant group, but the results showed increased heterogeneity. After using sensitivity analysis to remove the two studies with the greatest impact, it was found that the HAMD score was still not significantly changed in the EA group compared with the antidepressant group. Adverse events were reported in nine of the 19 studies included. Meta-analysis showed fewer adverse events in the EA group than in the antidepressant group, and there was no significant heterogeneity. Among them, one study [12] adopted the TESS score, revealing that the score of the EA group changed significantly compared to that of the antidepressant group. In summary, EA are effective in improving depressive symptoms. Compared with antidepressant medicine, EA has the advantages of fewer side events and better curative effects. In terms of safety, EA are superior to antidepressants.

Jadad ratings are displayed. Five studies [4, 5, 7, 10, 11] scored 3. Seven studies [1, 3, 13, 14, 16–18] scored 2. However, the rest of the studies [2, 6, 8, 9, 12, 15, 19] scored only 1. The quality evaluation of the included literature identified the following problems: (1) the random method was not rigorous enough or even missing: among all the included studies, nine studies used the randomized numerical tables. Five studies did not mention random sequence generation methods. The randomization principle in three studies was the order of visits. In two studies, computer software was used to generate random numbers for randomized grouping. (2) There is lack of allocation concealment. Of all the included literatures, only two studies mentioned allocation concealment. Both of these studies are assigned to test objects using opaque envelopes, which were not mentioned in the rest of the literatures. (3) Blinding methods were not used enough. None of the studies blinded the control group; only eight studies referred to blindness in outcome evaluation, whereas the rest of the literatures did not even describe it.

Although the results of this meta-analysis showed that EA was effective in improving PSD and its efficacy was not less than antidepressants. EA could also improve the living quality of PSD patients and promote the recovery of their neurological function. However, the quality of relevant clinical studies is still not high. In the future, high-quality RCT researches need to be conducted through strictly controlling random methods, allocation concealment, and blinding methods to provide a stronger support for the conclusions of the meta-analysis.

## Figures and Tables

**Table 1:** Main characteristics of included studies.

Study	Sample size	Diagnostic criteria of PSD	Baseline HAMD	Intervention	Control	No. of EA sessions	Treatment duration	Jadad score
Zhang and Yan [8]	E: 35 C: 35	CCMD/HAMD	E: 20.97 ± 4.16 C: 22.97 ± 5.77	GV20, HT7, LR3; 30 min, once a day	20 mg/d paroxetine	28	4	2 (2, 0, 0, 0)
Xu and Miago [7]	E: 40 C: 40	CCCD/CCMD/HAMD	E: 30.96 ± 4.25 C: 30.65 ± 3.98	GV20, EX-HN1, EX-HN3, LR3, HT7, PC6, KI3, SP6, BL15; 30 min, once a day	20 mg/d fluoxetine	42	6	1 (1, 0, 0, 0)
Wang et al. [6]	E: 25 E2: 25 E3: 25 C: 25	CCCD/CCMD/HAMD	E1: 22.35 ± 2.48 E2: 22.20 ± 1.97 E3: 21.97 ± 2.35 C: 21.38 ± 2.46	GV20, GV24, PC6, HT7; 30 min, once a day	50 mg/d sertraline	30	4	2 (1, 1, 0, 0)
Li et al. [19]	E: 11 C: 10	CCCD/HAMD	E: 31.2 ± 3.91 C: 30.2 ± 3.62	LI4, LR3; 20 min, once a day	20 mg/d fluoxetine	40	8	3 (2, 1, 0, 0)
Hong et al. [9]	E: 30 C: 30	CCCD/CCMD/HAMD	E: 25.45 ± 3.22 C: 24.87 ± 2.69	GV20, EX-HN1, GV24, ST36, LR3, SP6; 30 min, once a day	20 mg/d citalopram	30	4	3 (2, 1, 0, 0)
Kang and Yang [20]	E: 40 C: 40	CCCD/CCMD/HAMD	E: 36.83 ± 7.76 C: 35.17 ± 8.22	GV24, G13, GB31, SJ17, EX-HN3; 30 min, once a day	20 mg/d fluoxetine	28	4	1 (1, 0, 0, 0)
Huang et al. [10]	E: 30 C: 30	CCCD/CCMD/HAMD	E: 19.00 ± 2.55 C: 19.10 ± 2.46	GV20, GV18, G13 and G9; 30 min, once a day	20 mg/d fluoxetine	30	6	3 (2, 1, 0, 0)
Li et al. [21]	E: 35 C: 35	CCCD/CCMD/HAMD	E: 32.40 ± 3.92 C: 31.60 ± 4.37	EX-HN1, GV24, G13, LI4, LR3; 30 min, once a day	20 mg/d fluoxetine	28	4	1 (1, 0, 0, 0)
Zhu et al. [22]	E: 32 C: 30	CCCD/CCMD/HAMD	E: 26.20 ± 3.67 C: 27.10 ± 3.02	LI4, LR3; 20 min, once a day	20 mg/d fluoxetine	40	8	1 (1, 0, 0, 0)
Chen [11]	E: 47 C: 47	CCCD/CCMD/HAMD	E: 23.59 ± 4.04 C: 23.45 ± 3.69	GV20, EX-HN1, ST8, GB8, HT7; 30 min, once a day	20 mg/d fluoxetine	36	12	3 (2, 1, 0, 0)
Peng et al. [23]	E: 58 C: 59	CCCD/CCMD/HAMD	E: 30.19 ± 2.22 C: 29.87 ± 2.76	Nie San Zheng, PC6, ST36, LR3, ST40; 20 min, once a day	20 mg/d fluoxetine	24	4	3 (2, 1, 0, 0)
Guo et al. [12]	E: 32 C: 31	CCCD/CCMD/HAMD	E: 21.74 ± 5.50 C: 21.08 ± 5.72	GV20, EX-HN1, GV24, EX-HN3, LI4, LR3; 30 min, once a day	20 mg/d fluoxetine	30	6	1 (1, 0, 0, 0)
Zhou et al. [13]	E: 145 C:148	CCCD/CCMD/HAMD	E: 31.84 ± 13.12 C: 32.28 ± 12.57	GV20, EX-HN3, GV24, GV26, LI4, PC6, SP6, ST36, LR3; 30 min, once a day	20 mg/d fluoxetine	45	8	2 (2, 0, 0, 0)
Bi et al. [15]	E: 31 C: 32	CCCD/HAMD	E: 22.42 ± 2.93 C: 22.16 ± 2.16	GV20, EX-HN3; 45 min, once a day	20 mg/d fluoxetine	30	6	2 (2, 0, 0, 0)
Zhou [14]	E: 32 C: 34	CCCD/HAMD	E: 23.71 ± 9.36 C: 23.02 ± 10.47	GV20, EX-HN3; 45 min, once a day	20 mg/d fluoxetine	28	4	1 (1, 0, 0, 0)
Dong et al. [24]	E: 74 C: 34	CCCD/CCMD/HAMD	E: 24.67 ± 2.59 C: 24.12 ± 3.17	GV24, GV17, GB5, GB6, GV18, GB15, GB8, GB7, EX-HN3; 30 min, once a day	20 mg/d fluoxetine	30	4	2 (2, 0, 0, 0)
Chu et al. [16]	E: 36 C: 36	CCCD/CCMD/HAMD	E: 22.38 ± 5.87 C: 23.42 ± 6.12	GV20, EX-HN3, GV26, PC6; 30 min, once a day	20 mg/d fluoxetine	40	8	2 (2, 0, 0, 0)
Chen et al. [17]	E: 30 C: 30	CCCD/CCMD/HAMD	E: 29.34 ± 3.60 C: 31.05 ± 5.30	GV20, GV24, EX-HN1, EX-HN3, HT7, PC6, LR3; 20 min, once a day	20 mg/d fluoxetine	20	4	2 (2, 0, 0, 0)
Long et al. [18]	E: 36 C: 36	CCCD/HAMD	E: 22.30 ± 5.70 C: 22.70 ± 5.20	GV20, EX-HN3; 45 min, once a day	10-40 mg/d fluoxetine	24	4	1 (1, 0, 0, 0)

## Data Availability

All data are included in the manuscript.
